# Dopamine 2 receptor ablation from cholinergic neurons attenuates L-DOPA induced dyskinesias

**DOI:** 10.3389/fnagi.2026.1779861

**Published:** 2026-05-12

**Authors:** Santiago Uribe-Cano, Lauren Malave, Andreas H. Kottmann

**Affiliations:** 1 Department of Molecular, Cellular and Biomedical Sciences, CUNY School of Medicine, City University of New York, New York, NY, United States; 2 Neuroscience Collaborative, City University of New York Graduate Center, New York, NY, United States; 3 Department of Psychiatry, Columbia University, New York, NY, United States; 4 Molecular, Cellular and Developmental Subprogram, City University of New York Graduate Center, New York, NY, United States

**Keywords:** cholinergic interneurons, dopamine 2 receptor, dopamine 5 receptor, dyskinesias, L-dopa, Parkinson disease

## Abstract

**Introduction:**

L-DOPA-induced dyskinesia (LID) formation requires prominent dopamine oscillations over hours, across years of treatment. Striatal cholinergic interneurons (CIN) have been implicated in both the facilitation and attenuation of LID, but how CIN sense and respond to striatal dopamine levels in this context remains incompletely understood. CIN express both the inhibitory, Gα_i_-coupled dopamine D2 receptor and the facilitatory, Gα_s_-coupled dopamine D5 receptor. Although systemic ablation of D5 exacerbates LID, the contribution of CIN-specific D2 expression to LID has not been studied.

**Methods:**

We generated mice with conditional ablation of D2 from choline acetyltransferase-expressing cells (D2_ChAT_KO) and subjected them to unilateral 6-hydroxydopamine lesions followed by chronic L-DOPA dosing. We assessed dyskinetic behaviors across escalating L-DOPA doses and performed postmortem analyses of LID-associated signaling markers in dorsal striatal CIN. Specifically, we quantified p-ERK expression and phosphorylated rpS6^240/244^, a marker of translational activity, in CIN from mice examined in the L-DOPA ON and OFF states.

**Results:**

D2_ChAT_KO mice exhibited attenuated LID across escalating L-DOPA doses. Postmortem analyses suggested reduced expression of the LID-associated marker p-ERK among CIN of the dorsal striatum. In control mice, L-DOPA increased phosphorylated rpS6^240/244^ in a subset of CIN located in the dorsolateral striatum, indicating increased translational activity during the L-DOPA ON state. Ablation of D2 from CIN prevented this L-DOPA-associated increase in CIN phosphorylated rpS6^240/244^.

**Discussion:**

Together, these findings indicate that D2 signaling in CIN promotes LID formation and suggest that CIN D2 is a potential molecular target for mitigating dyskinesias while preserving the therapeutic efficacy of L-DOPA. These results are discussed in the context of recently refined models of how CIN may contribute to aberrant plasticity in the basal ganglia of the Parkinsonian mouse brain.

## 1 Introduction

Dopamine (DA) substitution therapy with the DA precursor L-3,4-dihydroxyphenylalanine (L-DOPA) remains the most efficacious treatment for the motor symptoms of Parkinson’s disease (PD) (Cotzias et al., 1969; Albin et al., 1989). However, most patients with advanced PD develop debilitating involuntary movements in response to this treatment known as L-DOPA-induced dyskinesias (LID). LID are thought to arise from slow oscillations in drug-associated DA levels (Bastide et al., 2015; de la Fuente-Fernandez et al., 2004; Zhai et al., 2025). Their development and expression require a hypodopaminergic state caused by degeneration of DA neurons (DAN) and consequent DA hypersensitivity, a condition that can be modeled in rodents and non-human primates (Cenci and Lundblad, 2007; Cenci et al., 2018). The resulting motor complications that present as LID are largely attributed to aberrant synaptic plasticity in striatal spiny projection neurons (SPN), the principal input neurons of the basal ganglia which is a central hub for motor learning (Cheung et al., 2023; Redgrave et al., 2010; Zhai et al., 2017). Under normal conditions, phasic elevations of DA from mesencephalic projections to the striatum reinforce actions associated with positive outcomes, whereas phasic reductions below tonic levels degrade actions associated with negative outcomes (Berke, 2018; Redgrave et al., 2010). Consistent, DAN degeneration and the resulting hypodopaminergia progressively impoverish movement through skewed motor learning that favors bradykinesia (Cheung et al., 2023). In contrast, the non-physiological, pulsatile DA surges produced by each L-DOPA dose, uncoupled from physiological action evaluation, may reinforce context-inappropriate motor programs that ultimately drive dyskinesias (Bastide et al., 2015; Cheung et al., 2023; Figge et al., 2024; Girasole et al., 2018; Ryan et al., 2024; Zhai et al., 2025; Zhuang et al., 2013).

In the healthy brain, striatal DA signaling is tightly regulated by ACh which is released from cholinergic interneurons (CIN) (Liu et al., 2022). CIN exhibit tonic pacemaker-like firing at 5–10 Hz and display complex phasic responses to salient stimuli, characterized by sustained pauses often flanked by burst firing that produces transient ACh fluctuations in the dorsolateral striatum (Aosaki et al., 1994b; Apicella et al., 2011; Duhne et al., 2024; Kimura et al., 1984; Krok et al., 2023; Morris et al., 2004; Ravel et al., 1999; Schulz and Reynolds, 2013; Zhang and Cragg, 2017). These cholinergic pauses develop during reinforcement learning and are anticorrelated with striatal DA release (Duhne et al., 2024; Morris et al., 2004). The molecular mechanisms underlying this “conditioned” pause have received considerable attention, in part because the resulting reductions in ACh are thought to create a temporal window during which DA can modify corticostriatal synapses on SPN (Aosaki et al., 1994a; Graybiel et al., 1994; Kimura et al., 1984; Morris et al., 2004; Wang et al., 2006).

In DA depleted animals, repeated L-DOPA bolus treatment distorts normal CIN activity both acutely and long term, producing elevated tonic firing, enhanced GABAergic input, abolition of the pause, and impaired learning (Aosaki et al., 1994a; Aosaki et al., 1994b; Cheung et al., 2023; Choi et al., 2020; Tubert et al., 2025; Zhai et al., 2025). Whether and how these CIN alterations are causal drivers of LID remains unclear (Bordia et al., 2016; Lim et al., 2014; Shen et al., 2022). Notably, both enhancement of cholinergic signaling and suppression via cyto-toxic ablation of CIN can attenuate LID (Ding et al., 2011; Nielsen and Ford, 2024; Shen et al., 2016; Won et al., 2014; Zhai et al., 2025). Nonetheless, L-DOPA clearly alters CIN activity, and CIN activity profoundly affects LID.

CIN cell-autonomous sensitivity to DA, and therefore to L-DOPA, is mediated by dopamine 2 receptor (D2) and dopamine 5 receptor (D5). This suggests that pathological CIN activity induced by L-DOPA may, at least in part, reflect altered signaling through one or both receptors (Jarvie et al., 1993; Tiberi and Caron, 1994; Tiberi et al., 1996; Yan et al., 1997). Supporting this possibility, systemic ablation of the medium-affinity, high-constitutive activity D5 (Tiberi et al., 1991), a Gα_s_-coupled GPCR (Grandy et al., 1991), facilitates LID (Castello et al., 2020). Specifically, D5 knockout mice displayed exacerbated abnormal involuntary movements (AIMs) that worsened with repeated L-DOPA dosing. Although these studies relied on global ablation of D5, its restricted expression in other striatal cell types supports a CIN-specific mechanism (Yan and Surmeier, 1997). Consistent, inverse agonism of D5 with clozapine restores the cholinergic pause in L-DOPA-treated parkinsonian mice by relieving Kv1 channels from tonic D5 inhibition in CIN (Tubert et al., 2025).

In contrast, the role of Gα_i_-coupled, high-affinity D2 in CIN during LID is poorly defined. In the healthy brain, D2 activation regulates multiple intrinsic conductances, suppresses autonomous CIN spiking, and reduces ACh release from axon terminals (Chantranupong et al., 2023; Chuhma et al., 2014; Ding et al., 2006; Straub et al., 2014; Wieland et al., 2015). Cell type-specific loss- and gain-of-function studies further show that D2 signaling modulates cholinergic pause duration in a graded manner (Gallo et al., 2022;Kharkwal et al., 2016; Martyniuk et al., 2022). This D2-dependent modulation of CIN activity is critical for DA-dependent striatal plasticity (Wang et al., 2006). However, despite its clear role in physiological striatal plasticity, whether this mechanism contributes to aberrant plasticity in LID remains untested.

Given the opposing regulation of CIN physiology by D2 and D5, and our previous findings that peptidergic signaling via the GPCR smoothened on CIN attenuates LID by opposing D2 (Malave et al., 2021), we hypothesized that D2 signaling in CIN represents a molecular mechanism by which L-DOPA facilitates LID. Consistent with this idea, selective ablation of D2 from ChAT^+^ cells (D2_ChAT_KO) in a 6-OHDA LID model attenuated dyskinesias and prevented expression of the LID-associated marker p-ERK in CIN. D2_ChAT_KO also appeared to attenuate L-DOPA-associated increases in the translation activity marker p-rpS6^240/244^. Together, these findings raise the possibility that removal of D2 receptors from CIN rebalances ACh tone in the L-DOPA–treated PD brain, thereby restoring balanced DA–ACh signaling at downstream SPNs. These results therefore warrant corroboration in additional genetic and non-human primate models of LID.

## 2 Materials and methods

### 2.1 Anesthesia

Anesthesia was induced using a gas mixture of oxygen (1.5 L/min) and 4% isoflurane (Covetrus).

### 2.2 Euthanasia

Euthanasia was achieved by terminal transcardial perfusion. Mice were deeply anesthetized with intraperitoneal pentobarbital (15 Mg/Kg) and perfused transcardially with 0.9% saline followed by 4% paraformaldehyde (PFA) in 0.1 M phosphate-buffered saline (PBS).

### 2.3 Animals

Mice were bred on a C57BL/6 J background. All experiments were performed in adult mice between 3 and 6 months of age. Both sexes were included and balanced across experimental conditions, along with age. Cholinergic-specific allele recombination was driven by Cre expression under the control of the choline acetyltransferase promoter (ChATCre^+/−^) (Rossi et al., 2011). Conditional ablation of D2 was achieved using a floxed D2 allele (Bello et al., 2011). Experimental animals were generated from a D2^L/+^: ChATCre^+/−^ × D2^L/+^ cross, following an initial ChATCre^+/−^ × D2^L/L^ pairing. Homozygous D2^L/L^: ChATCre^+/−^ animals (D2_ChAT_KO) lacked D2 in ChAT^+^ cells, among which CIN are included. Controls were ChATCre^+/−^ or heterozygous D2^L/+^: ChATCre^+/−^ mice.

### 2.4 6-OHDA lesion and L-DOPA treatment

To induce a Parkinsonian state, mice received unilateral intrastriatal 6-OHDA (Sigma-Aldrich H4381) at a concentration of 3.0 Mg/Ml, dissolved in saline containing 0.2 g/L ascorbic acid (Sigma-Aldrich A92902). This approach, combined with the L-DOPA dosing schedule outlined below, has shown to produce robust LID alongside significant DAN denervation (Bastide et al., 2015; Girasole et al., 2018; Lundblad et al., 2004). The 6-OHDA solution was kept on ice, protected from light, and prepared fresh every 3 h during surgeries. Injections were performed in the dorsolateral (DL) striatum under anesthesia as specified above. Two unilateral injections (2 × 2 μL each) of 6-OHDA solution were administered into the left striatum relative to bregma at coordinates anteroposterior (AP): +1.0 mm, mediolateral (ML): +2.1 mm, dorsoventral (DV): −2.9 mm; and AP: +0.3 mm, ML: +2.4 mm, DV: −2.9 mm. Injections were performed using a Hamilton syringe, attached to a micro-syringe pump, at a rate of 0.4 μL/min. The syringe was left in place for 3 min following infusion and then retracted slowly to minimize backflow. A minimum of 18 days was allowed post-surgery to ensure sufficient degeneration of DAN before onset of behavioral testing.

After the recovery period, Parkinsonian phenotype was confirmed via open-field turn bias and cylinder paw-use tests. Open field testing was performed in a 43 × 43 cm open-field arena (ENV 515S; Med Associates), and cylinder tests were performed in a large 2 L glass beaker.

L-DOPA (5–20 Mg/Kg) was administered daily in the home cage, except on days when behavioral testing occurred. Therapeutic response was assessed after the first dose using the open-field and cylinder tests. L-DOPA dosing escalated as follows: 5 Mg/Kg (Days 1–13), 10 Mg/Kg (Days 14–16), 15 Mg/Kg (Days 17–18), and 20 Mg/Kg (Days 19–31). After 31 days, half the animals received 9 g/L saline for 1 week (washout), while the other half continued L-DOPA treatment (20 Mg/Kg) for one week. Animals were sacrificed 30 min after the final dose for immunohistochemical analyses with pERK, p-rpS6^240/244^ and Tyrosine Hydroxylase (TH). A 30 min interval was chosen to make the results of these analyses comparable with previous literature (Bastide et al., 2015; Castello et al., 2020; Malave et al., 2021). Mice that failed to show at least an 80% reduction in the dopaminergic marker TH in the lesioned DL striatum were excluded (2 controls, 1 mutant). This threshold is consistent with validated levels of reduction in TH fiber density in the LID literature (Bastide et al., 2015; Cortes et al., 2017; Malave et al., 2021; Pelosi et al., 2023).

### 2.5 Dyskinesia quantification

Abnormal involuntary movements (AIMs) were regularly quantified throughout the dosing regimen. On assessment days, animals received the specified L-DOPA dose offset from each other by one-minute intervals and were placed individually in empty housing cages without bedding. At 20-min intervals, over a total duration of 2 h, each animal was serially observed for a one-minute period. During each observation, the total number of contralateral rotations and the severity of AIMs were recorded in accordance with validated methods (Sebastianutto et al., 2016). AIM severity was scored for each of the defined subtypes (axial, limb, orofacial) for each one-minute interval on a scale of 0–4: 0 = no expression of the subtype; 1 = expression for less than 50% of the observation period; 2 = expression for more than 50% of the observation period; 3 = continuous but interruptible expression throughout the observation period; 4 = continuous and uninterruptible expression throughout the observation period. AIM scores across the two-hour session were summed for each mouse to yield a total AIM score. Data were collected from 3 independent cohorts: 1 utilizing heterozygous D2^L/+^: ChATCre^+/−^ controls and 2 utilizing ChATCre^+/−^controls. All behavioral testing, as well as subsequent immunohistochemical data collection and analysis, was performed by experimenters blinded to genotype.

### 2.6 Immunohistochemistry

Mice were deeply anesthetized with intraperitoneal pentobarbital (15 Mg/Kg) and perfused transcardially with 0.9% saline followed by 4% paraformaldehyde (PFA) in 0.1 M phosphate-buffered saline (PBS). Brains were post-fixed overnight at 4 °C in 4% PFA, then cryoprotected in 30% sucrose in PBS until they sank (~48 h). Cryoprotected brains were embedded in optimal cutting temperature (OCT) compound, frozen on dry ice, and stored at −80 °C. Brains were cryo-sectioned coronally at 30 μm using a cryostat at −20 °C and stored as free-floating sections in PBS with 0.1% sodium azide until staining.

Anti-TH (1:500; RRID: AB_657012) and anti-ChAT (1:100; RRID: AB_144P) were purchased from Millipore. Anti-p-ERK1/2 (1:400; RRID: AB_9101), and anti-p-rpS6^240/244^ (1:800; RRID: AB_5364) were purchased from Cell Signaling Technology. Anti-NeuN (1:200; RRID: AB_MAB377) was purchased from Chemicon International. Various Alexa Fluor antibodies were used at 1:250 dilutions for immunohistochemistry (Jackson Immuno Research).

Images of fluorescent immunoreactivity used for quantification were acquired using a ZEISS LSM 880 confocal microscope. Images were taken at 20 × magnification (10 × for assessing 6-OHDA-induced DAN axon loss). All images taken were single optical planes. Confocal fluorescence was quantified via ImageJ. For loss of dopaminergic innervation in the striatum, TH Mean Gray Value (MGV) was measured across the entire striatum and normalized to background. Percent TH was calculated relative to the unlesioned hemisphere. Lesion extent was mapped across multiple striatal/midbrain sections (Supplementary Figure 2). For p-ERK and p-rpS6^240/244^ analysis, 700um^2^ fields centered at DL (ML: +/−2.2 mm; DV: −3.0 mm) or dorsomedial (DM) (ML: +/−1.2 mm; DV: −2.5 mm) positions and spanning AP: 0.3-1 mm were used.

For p-ERK, MGV was measured in CIN (ChAT^+^ cells) of both hemispheres. CIN were considered p-ERK^+^ if their MGV exceeded the unlesioned hemisphere mean +2 SD. Percentage of p-ERK^+^ CIN was reported per lesioned striatum. The same procedure was used for p-ERK^+^/NeuN^+^ cells (excluding ChAT^+^ cells).

For p-rpS6^240/244^, MGV was calculated for ChAT^+^ regions of interest (ROIs) and normalized to NeuN MGV within the same ROI.

## 3 Results

### 3.1 Validation of D2_ChAT_KO 6-OHDA model

**Figure 1:**
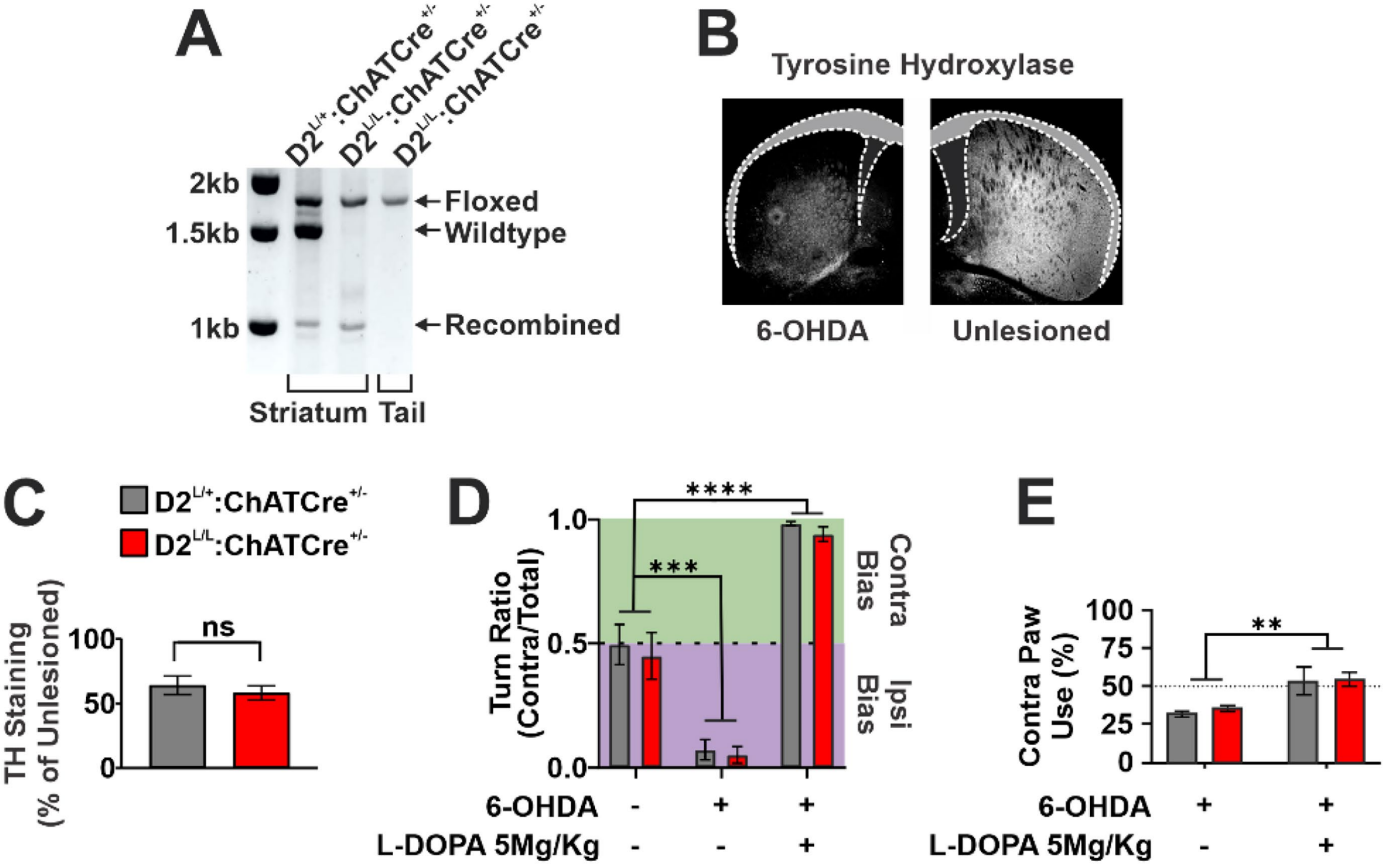
**(A)** PCR validation of ChATCre-dependent recombination of the dopamine 2 receptor (D2) allele. Primers amplify regions spanning exon 2 and the flanking LoxP sites. Absence of a recombined band in tail samples confirms recombination is restricted to ChATCre-expressing cells, including cholinergic interneurons (CIN) of the striatum. **(B)** Representative image showing tyrosine hydroxylase (TH) depletion in the dorsal striatum following 6-hydroxydopamine (6-OHDA) injection. **(C)** Quantification of TH depletion in the lesioned dorsal striatum, expressed as the lesioned/unlesioned hemisphere ratio, shows no difference between control and D2^L/L^: ChATCre^+/−^ animals (*n* = 5–7 per genotype; unpaired two-tailed Student’s *t* test, *p* > 0.05). **(D)** Turn bias in the open field, measured at baseline, after unilateral 6-OHDA lesion, and after subsequent L-DOPA treatment (5 Mg/Kg). Turn bias was quantified as the ratio of contralateral turns over total turns. Both genotypes showed comparable reversal of 6-OHDA-induced turn bias following L-DOPA treatment (*n* = 5–7 per genotype; mixed-effects model: Timepoint effect, *F*(1.220, 17.69) = 109.7, *p* < 0.0001; Genotype effect, *F*(1, 29) = 0.55, *p* > 0.05; Timepoint × Genotype interaction, *F*(2, 29) = 0.03, *p* > 0.05; *post hoc* Tukey’s multiple comparisons test: ^***^p < 0.001, ^****^p < 0.0001). **(E)** Cylinder test measuring the percentage of paw rears performed with the forelimb contralateral to the lesion, before and after L-DOPA treatment. Both genotypes exhibited similar L-DOPA-dependent increases in contralateral paw use (*n* = 5–7 per genotype; two-way repeated-measures ANOVA: Timepoint effect, *F*(1, 10) = 20.67, *p* < 0.01; Genotype effect, *F*(1, 10) = 0.21, *p* > 0.05; Timepoint × Genotype interaction, *F*(1, 10) = 0.08, *p* > 0.05).

To selectively ablate D2 from CIN, we generated mice homozygous for a floxed D2 allele (D2^L/L^) expressing Cre recombinase under the choline acetyltransferase (ChAT) promoter (D2^L/L^: ChATCre^+/−^). These mice lack D2-mediated electrophysiological and cholinergic biosensor responses to DA and D2 agonists in CIN as well as fail to exhibit attenuated cholinergic pauses following D2 antagonism (Martyniuk et al., 2022). Importantly, D2 expression is preserved in other striatal cell types, including D2-expressing SPN (Augustin et al., 2018). Consistent with this specificity, prior analyses demonstrated that this genetic strategy selectively reduces D2 mRNA in ChAT^+^ cells within striatal tissue (Augustin et al., 2018). In the present study, successful recombination of the D2 locus was confirmed in striatal, but not tail, tissue (Figure 1A). Littermate controls heterozygous for the floxed allele and carrying the ChATCre driver (D2^L/+^: ChATCre^+/−^) were used to control for both Cre expression and floxed allele insertion.

Following intrastriatal 6-OHDA injections targeting the DL striatum, both D2^L/L^: ChATCre^+/−^ and control animals exhibited similar levels of striatal DAN degeneration, indicating that D2 deletion from CIN does not alter 6-OHDA-induced cytotoxicity (Figures 1B,C).

To validate the Parkinsonian phenotype, we employed two standard assays of unilateral motor dysfunction. In the open field, 6-OHDA lesions induced a robust ipsilateral turning bias (Figure 1D). Similarly, the cylinder test revealed an ipsilateral forelimb-use preference, confirming equivalent hypodopaminergic motor deficits across genotypes (Figure 1E).

Subsequent administration of 5 Mg/Kg L-DOPA reversed these motor asymmetries in both assays: turning bias in the open field shifted contralaterally, and forelimb use in the cylinder test similarly favored the contralateral paw (Figures 1D,E). These effects were equivalent in control and D2^L/L^: ChATCre^+/−^ animals, indicating that deletion of D2 from CIN does not impair the prokinetic efficacy of L-DOPA.

### 3.2 Loss of D2 on ChAT^+^ cells attenuates L-DOPA induced dyskinesias

Treatment with L-DOPA over the course of the experiment employed a chronic dosing schedule that escalated over the course of 31 days, after which, half the animals from each genotype underwent a 1-week washout period during which they received saline while the other half continued L-DOPA dosing at the highest dose of 20 Mg/Kg. The rationale for this design was twofold: First, p-ERK, a validated histological marker of LID severity, implicates CIN as critical contributors in later stages of LID (Ding et al., 2011). We therefore aimed to capture these progressive mechanisms with an extended dosing regimen. Second, we sought to assess how CIN, with or without D2 expression, responded to L-DOPA once LID was fully established. By isolating the contribution of D2 signaling to the acute induction of LID at this stage, we hoped to gain insight into the mechanisms that sustain LID once developed, independent of the early-stage plasticity likely prominent during initial L-DOPA treatment.

**Figure 2:**
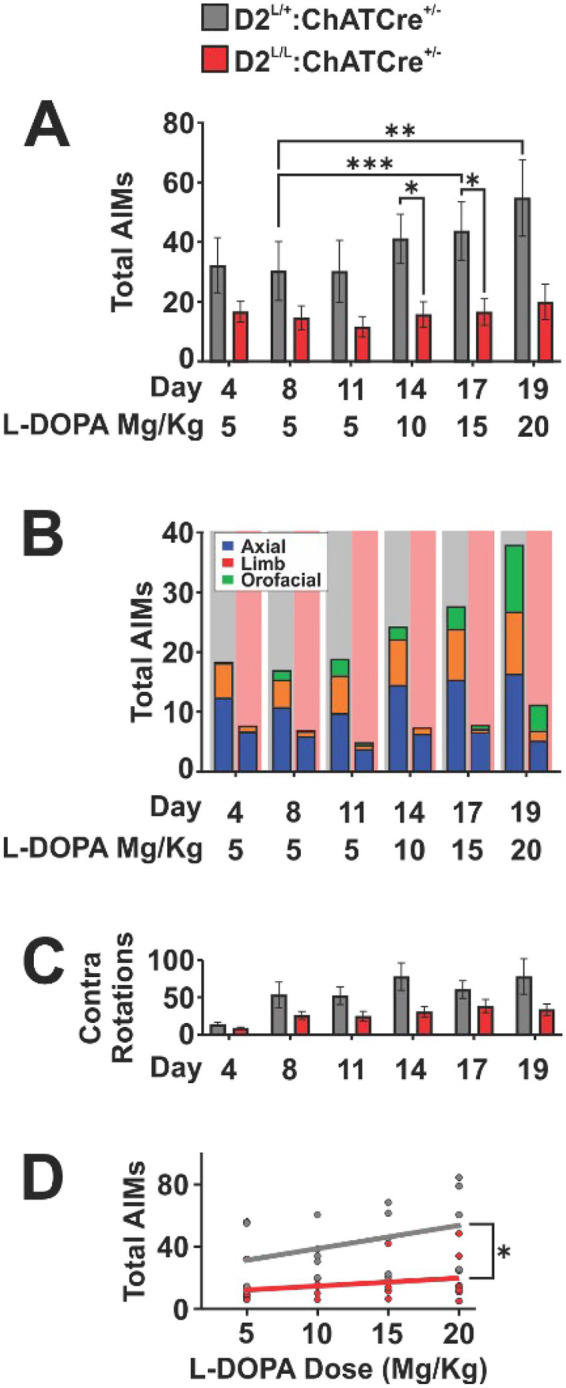
**(A)** Total abnormal involuntary movement (AIM) scores over the first 19 days of L-DOPA treatment (*n* = 5–7 per genotype; two way repeated-measures ANOVA: Day/Dose effect, *F*(2.462, 24.62) = 11.29, *p* < 0.001; Genotype effect, *F*(1, 10) = 6.03, *p* < 0.05; Genotype × Day/Dose interaction, *F*(5, 50) = 4.50, *p* < 0.01; post hoc Tukey’s multiple comparisons test: ^*^p < 0.05, ^**^p < 0.01, ^***^p < 0.001). **(B)** Breakdown of AIM subtypes across L-DOPA treatment days. Stacked bars represent the average group values for each AIM subtype; column background indicates genotype. **(C)** Total contralateral rotations during AIM scoring sessions across the first 19 days of L-DOPA treatment (*n* = 5–7 per genotype; two-way repeated-measures ANOVA: Day/Dose effect, *F*(2.311, 23.11) = 15.92, *p* < 0.0001; Genotype effect *F*(1, 10) = 4.75, *p* > 0.05; Genotype × Day/Dose interaction, *F*(5, 50) = 3.53, *p* < 0.01). **(D)** AIM severity plotted against L-DOPA dose for each genotype. Slope differences assessed by linear regression followed by ANCOVA (*n* = 5–7 per genotype; *F*(1, 4) = 15.97, *p* < 0.05).

Control animals displayed persistent AIMs that worsened as L-DOPA dose increased on days 14, 17, and 19 (Figure 2A). In contrast, D2_ChAT_KO mice exhibited consistently attenuated AIMs, with the largest differences from controls observed at higher doses. Reductions across axial, limb, and orofacial AIM subtypes accounted for the lower total AIM scores in D2_ChAT_KO animals (Figure 2B; see Supplementary Video).

When comparing total contralateral rotations between genotypes on AIM scoring days, we observed an interaction effect. While there was no difference in rotation bias in the open field on day 4 of L-DOPA treatment, control animals developed a progressive increase in rotational bias over time (Figure 2C).

LID severity is known to be associated with the strength of L-DOPA dose administered. To see if this correlation was impacted by D2 ablation from cholinergic neurons, we performed linear regression of total AIM severity across the four L-DOPA doses tested for both controls and D2_ChAT_KO animals. The resulting slopes differed significantly between genotypes, indicating that the positive correlation between L-DOPA dose and AIM severity observed in controls was attenuated among D2^L/L^: ChATCre^+/−^ animals. This analysis further supported the attenuation of LID escalation in D2^L/L^: ChATCre^+/−^ mice compared to controls (Figure 2D).

To address potential gene dosage effects in heterozygous controls, we replicated these behavioral experiments using pooled data from independent cohorts with alternative ChATCre^+/−^controls carrying two wild-type D2 alleles (Supplementary Figure 1). In this replication cohort, AIMs again increased as a function of dose, confirming LID. Although Genotype and Genotype × Day interaction effects did not reach significance in this analysis, exploratory *post hoc* comparisons detected reduced AIMs in D2^L/L^: ChATCre^+/−^ mice at select doses (Supplementary Figures 1D,G), qualitatively consistent with the attenuated escalation of LID previously observed.

To exclude the possibility that reduced dyskinesias reflected differences in lesion severity among the replication cohort, we performed detailed mapping of TH loss in the striatum and midbrain of both genotypes (Supplementary Figure 2). This analysis revealed substantial reductions in striatal dopaminergic neurite density and corresponding midbrain DAN cell body density in the 6-OHDA-treated hemispheres of both mutants and controls. While this analysis rules out the effects of inadequate lesioning in our replication cohort, it does not address potential differences in lesion severity between the initial and replication cohorts which might explain differences in the strength of the observed effect.

### 3.3 Reduced CIN expression of p-ERK reflects attenuated AIMs among D2_ChAT_KO animals

Phosphorylation of extracellular signal-regulated kinase1/2 (p-ERK) in the hypodopaminergic striatum reflects LID severity and progression (Ding et al., 2011; Malave et al., 2021; Won et al., 2014). Rodent models of LID have shown that p-ERK expression occurs in both SPN and CIN and is correlated with severity of dyskinesias. Notably, early-stage L-DOPA treatment primarily increases p-ERK in SPN, while chronic L-DOPA exposure shifts this expression to CIN (Ding et al., 2011; Won et al., 2014). Moreover, pharmacological inhibition of p-ERK has been shown to attenuate LIDs, suggesting that p-ERK activation may not only reflect, but also contribute to LID pathophysiology (Ding et al., 2011). We therefore assessed whether the differences in LID severity between heterozygous controls and D2^L/L^: ChATCre^+/−^ animals were reflected in p-ERK expression at the end of our chronic L-DOPA dosing regimen.

**Figure 3:**
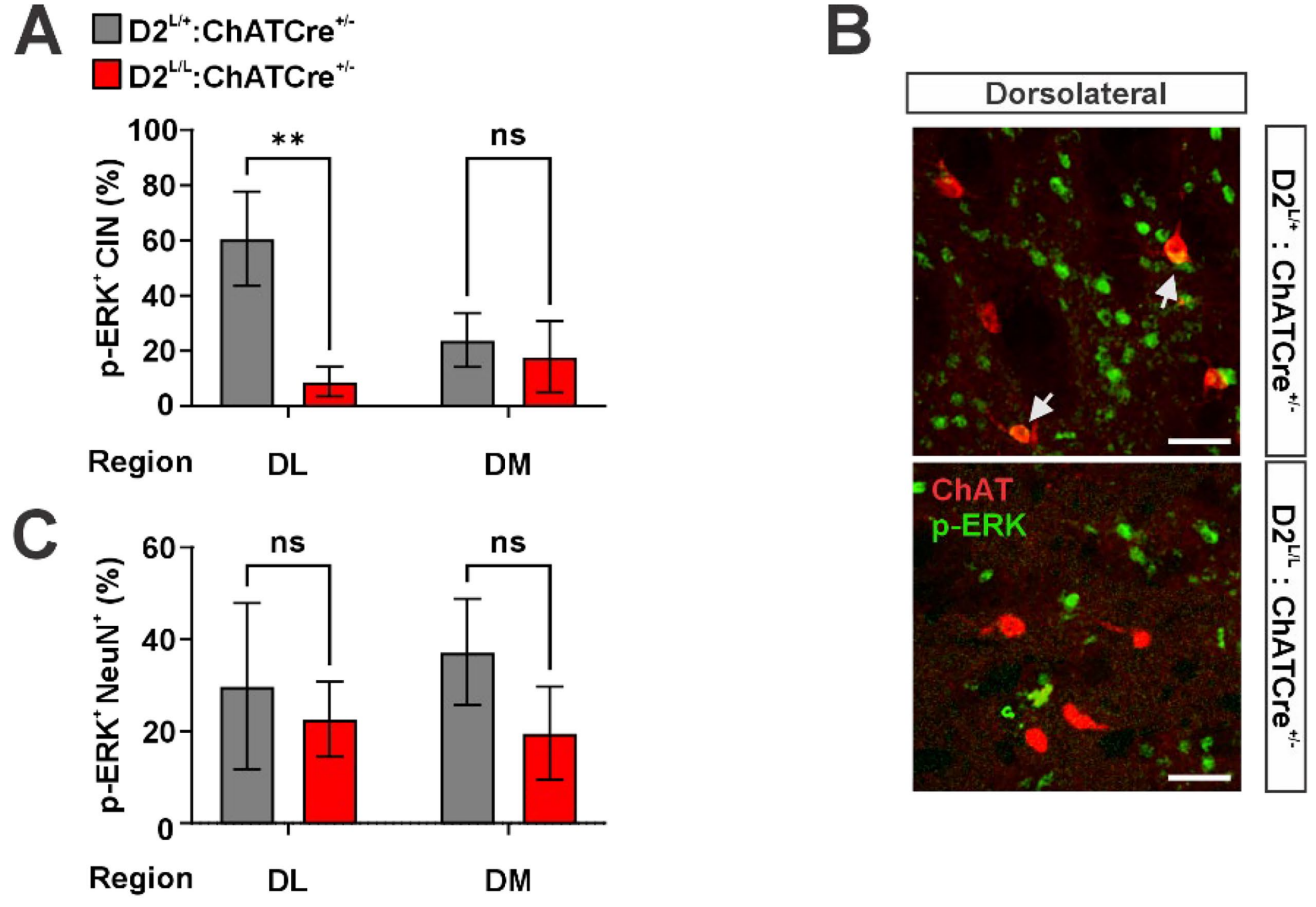
**(A)** Quantification of phosphorylated extracellular signal-regulated kinase 1/2 positive (p-ERK^+^) cholinergic interneurons (CIN), reported as the percentage of total CIN in the dorsolateral (DL) or dorsomedial (DM) striatum (*n* = 2–3 striatal sections per animal, across 2–3 animals per genotype; two-way repeated-measures ANOVA: Genotype effect, *F*(1, 12) = 5.96, *p* < 0.05; Region effect, *F*(1, 12) = 1.45, *p* > 0.05; Genotype × Region interaction, *F*(1, 12) = 3.96, *p* = 0.069; post hoc Šídák’s multiple comparisons test: ^**^p < 0.01). **(B)** Representative images of p-ERK and choline acetyltransferase (ChAT) immunoreactivity in the DL striatum (scale bar = 50 μm). **(C)** Quantification of p-ERK^+^/ChAT^−^ neurons, reported as the percentage of total ChAT^−^ neurons in the DL or DM striatum (*n* = 2–3 striatal sections per animal, across 2–3 animals per genotype; two-way repeated-measures ANOVA: Genotype effect, *F*(1, 12) = 0.05, *p* > 0.05; Region effect, *F*(1, 12) = 0.85, *p* > 0.05; Genotype × Region interaction, *F*(1, 12) = 0.27, *p* > 0.05).

Consistent with the behavioral findings, D2^L/L^: ChATCre^+/−^ animals exhibited a significant reduction in p-ERK^+^ CIN across the striatum (Figures 3A,B). While the Genotype × Striatal Region interaction effect only approached significance, exploratory post hoc comparisons showed a significant genotype difference specifically in the DL striatum, a qualitative effect consistent with the DL striatum’s predominant role in LID formation and expression (Girasole et al., 2018).

When we examined p-ERK expression in non-cholinergic NeuN^+^ neurons of the striatum, which are mostly SPN, we found no genotype differences, consistent with the chronic nature of the L-DOPA dosing paradigm and the cholinergic neuron specificity of D2 ablation (Figure 3C).

### 3.4 D2_ChAT_KO prevents L-DOPA-induced increases in CIN-specific p-rpS6^240/244^

**Figure 4:**
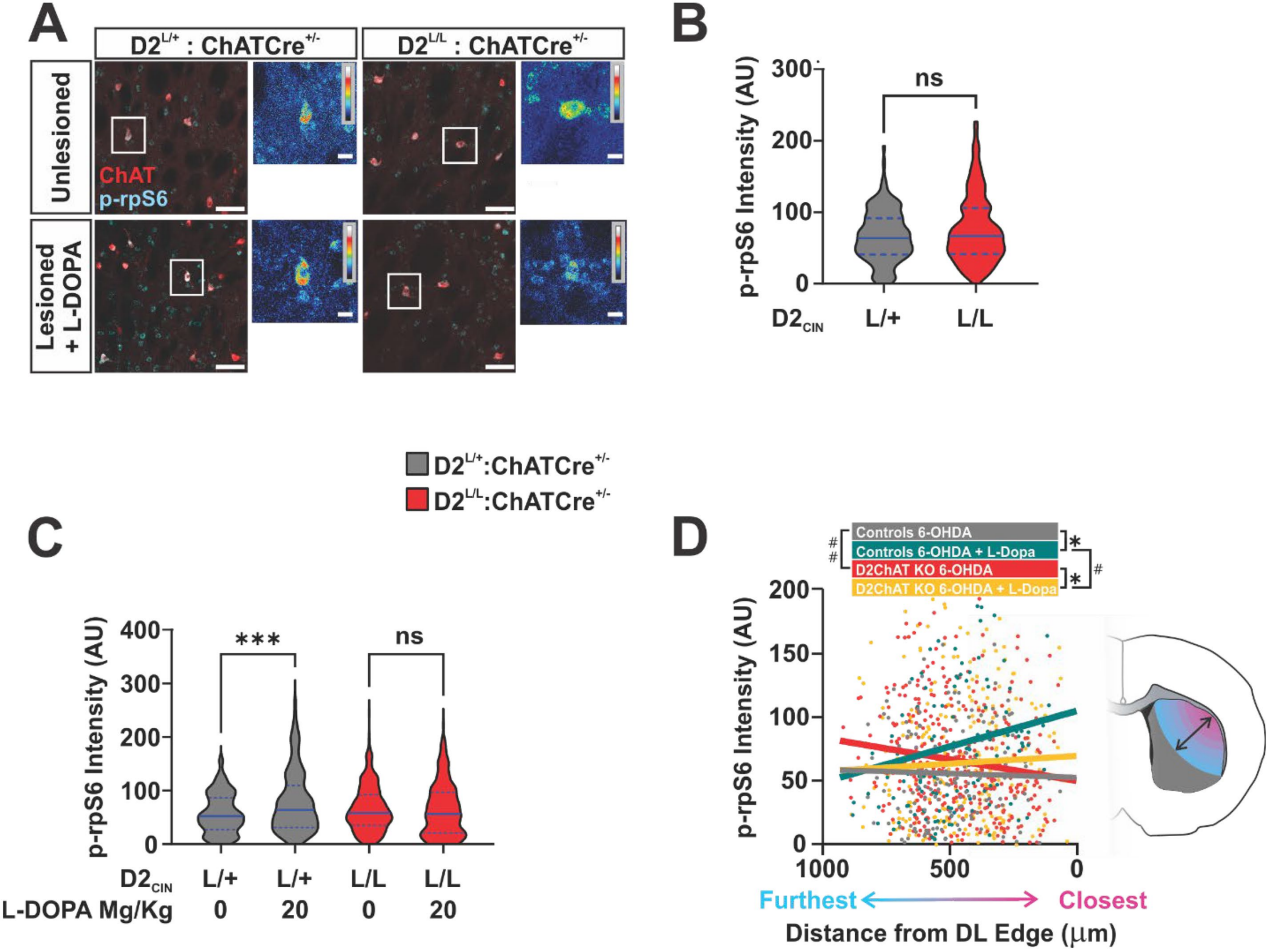
**(A)** Representative images from each genotype showing phosphorylated ribosomal protein S6 (p-rpS6^240/244^) and choline acetyltransferase (ChAT) immunoreactivity in the dorsolateral (DL) striatum under two conditions: unlesioned hemispheres (no 6-OHDA, L-DOPA washout) and lesioned hemispheres following L-DOPA treatment (scale bar = 50 μm). White rectangles indicate inset regions displaying heat maps of p-rpS6^240/244^ intensity within individual CIN (inset scale bar = 5 μm). **(B)** Quantification of normalized CIN p-rpS6^240/244^ intensity in the DL striatum of unlesioned hemispheres (no 6-OHDA, L-DOPA washout; *n* = 150 + CIN sampled from 4 striatal sections per animal, across 2–3 animals per genotype; unpaired two tailed Student’s *t* test, *p* > 0.05). **(C)** Quantification of normalized CIN p-rpS6^240/244^ intensity in the DL striatum of 6-OHDA-lesioned hemispheres with and without L-DOPA washout (*n* = 150 + CIN sampled from 4 striatal sections per animal, across 2–3 animals per genotype; two way ANOVA: Genotype effect, *F*(1, 957) = 0.26, *p* > 0.05; L-DOPA effect, *F*(1, 957) = 8.29, *p* < 0.01; Genotype × L-DOPA interaction, *F*(1, 957) = 11.90, *p* < 0.001; *post hoc* Fisher’s LSD: ^***^p < 0.001). **(D)** Plot of individual DL CIN p-rpS6^240/244^ intensities across distances from the DL edge of the striatum color-coded by condition. Striatal diagram demonstrates dorsolateral-to-medioventral axis along which distance from the DL edge was calculated for each CIN. Best-fit lines for each condition are plotted (*n* = 150 + CIN sampled from 4 striatal sections per animal, across 2–3 animals per condition). Comparison of all four best fit lines revealed significantly different slopes (slope comparison ANCOVA: *F* [3, 953] = 4.91, *p* = 0.0022). Individual pairwise ANCOVA comparisons were also performed to find specific differences between conditions. Asterisks denote significantly different slopes while hashmarks denote significantly different y-intercepts (Controls 6-OHDA vs. Controls 6-OHDA + L-DOPA slope comparison ANCOVA: *F* [1, 283] = 4.09, ^*^p < 0.05; D2_ChAT_KO 6-OHDA vs. D2_ChAT_KO 6-OHDA + L-DOPA slope comparison ANCOVA: *F* [1, 670] = 6.00, ^*^p < 0.05; Controls 6-OHDA vs. D2_ChAT_KO 6-OHDA slope comparison ANCOVA: *F* [1, 538] = 1.51, *p* = 0.22; Controls 6-OHDA vs. D2_ChAT_KO 6-OHDA intercept comparison ANCOVA: *F* [1, 539] = 7.16, ^##^*p* < 0.001; Controls 6-OHDA + L-DOPA vs. D2_ChAT_KO 6-OHDA + L-DOPA slope comparison ANCOVA: *F* [1, 415] = 2.05, *p* = 0.15; Controls 6-OHDA + L-DOPA vs. D2_ChAT_KO 6-OHDA + L-DOPA intercept comparison ANCOVA: *F* [1, 416] = 6.33, ^#^*p* < 0.05).

To assess D2’s impact on CIN activity during LID, we quantified CIN-specific levels of p-rpS6^240/244^ (Figure 4A). Phosphorylation of rpS6^240/244^ serves a marker of translational activity and has been associated with changes in overall CIN excitability under certain contexts (Bertran-Gonzalez et al., 2012; Matamales et al., 2016). Focusing on the established LID state, we compared CIN in the DL striatum of control and D2_ChAT_KO mice following continued L-DOPA treatment (L-DOPA ON) or after a 1-week washout period (L-DOPA OFF). Because no differences between genotypes were observed in the unlesioned hemispheres of L-DOPA OFF animals, we assumed that D2_ChAT_KO had no relevant baseline effects on p-rpS6^240/244^ expression (Figure 4B).

Likewise, CIN of the lesioned hemisphere in L-DOPA OFF animals showed no genotype difference (Figure 4C; columns 1 vs. 3). However, in lesioned hemispheres of L-DOPA ON animals, CIN p-rpS6^240/244^ levels were increased in controls but not D2_ChAT_KO mice (Figure 4C; columns 2 vs. 4). Anatomically, this change in p-rpS6^240/244^ levels was mediated by dorsolateral-most CIN. Linear regression relating each CIN’s p-rpS6^240/244^ value to its distance from the DL edge of the striatum revealed that CIN translational activity was relatively uniform across the striatum of control 6-OHDA animals in the L-DOPA OFF state (Figure 4D; Gray). However, L-DOPA treatment introduced a spatial bias in p-rpS6^240/244^ levels, such that dorsolateral-most CIN exhibited relatively higher values than those located more medially (Figure 4D; Comparison of Gray vs. Green Slopes). In contrast, L-DOPA exposure did not produce this DL enrichment of p-rpS6^240/244^ in D2_ChAT_KO animals (Figure 4D; Red vs. Yellow). Together, these data suggest a trend in which chronic L-DOPA exposure promotes a relative elevation of p-rpS6^240/244^ levels and ERK phosphorylation in CIN of the DL striatum through a cell-autonomous D2-dependent mechanism. Conversely, ablation of D2 from cholinergic neurons prevents this shift and attenuates AIMs.

## 4 Discussion

We find that conditional ablation of D2 from ChAT^+^ neurons attenuates LID in the unilateral 6-OHDA model, with the clearest separation from controls emerging at higher L-DOPA doses. This anti-dyskinetic effect is accompanied by reduced pERK in CIN and loss of an L-DOPA ON–associated increase in CIN p-rpS6^240/244^ among a DL CIN subpopulation. These effects occur despite comparable lesion severity and preserved prokinetic efficacy of L-DOPA in mutants and controls, suggesting that CIN D2 signaling might contribute to dyskinesia-related circuit remodeling and/or L-DOPA ON/OFF state transitions rather than to the beneficial motor response to L-DOPA.

Our study has several limitations and should be corroborated in multiple independent models of LID further discussed below. To facilitate the formulation of expectations in these future studies, we attempt here to integrate our observations with recently refined models of LID and reinforcement learning, as well as suggest follow up experiments.

### 4.1 LID is a complication caused by aberrant temporal coordination of DA and ACh oscillations

**Figure 5:**
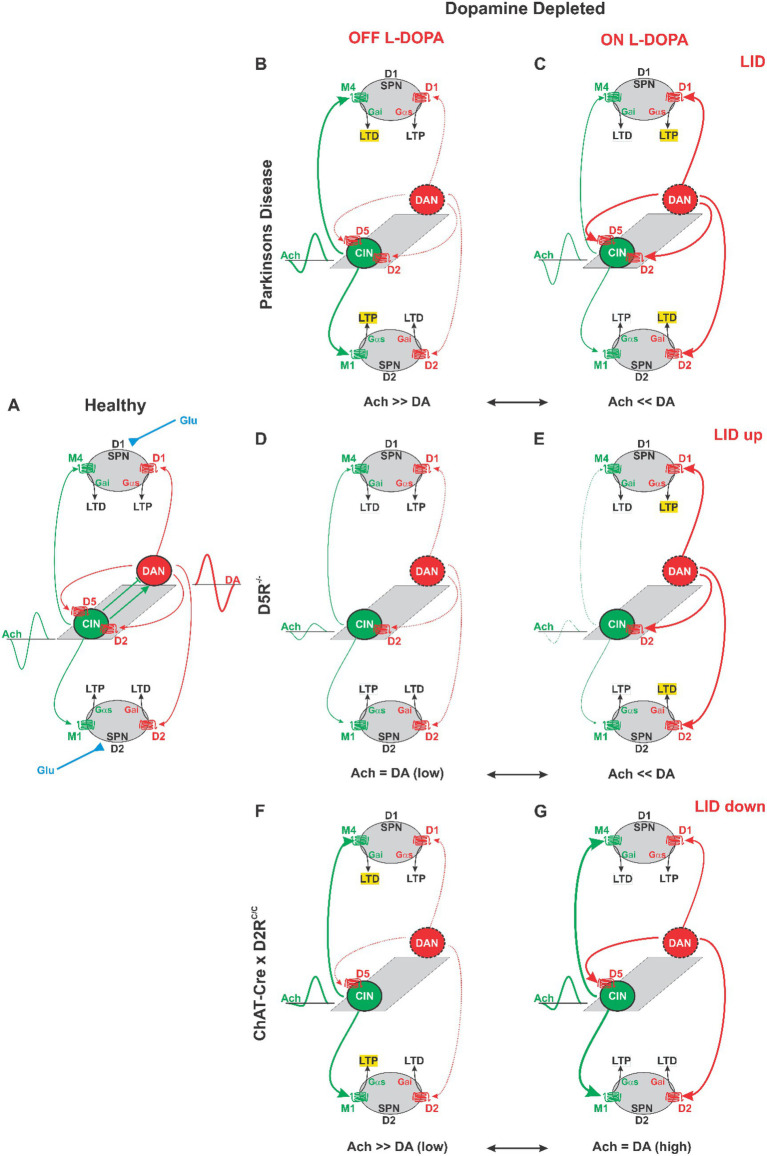
Integrated working model of reciprocal cholinergic (green) and dopaminergic (red) control of striatal plasticity across levodopa OFF/ON states, and predicted consequences of CIN-D5 and CIN-D2 ablation for LID. Line thickness indicates the relative influence of each neuromodulator; yellow fill denotes biased corticostriatal plasticity (LTP and LTD) in direct-pathway SPNs (dSPNs) and indirect-pathway SPNs (iSPNs). Dopaminergic axon degeneration is indicated by a dotted red line; red arrows scale with DA availability in OFF and ON states. DAN and CIN influence each other reciprocally (grey plane in center of each panel). For clarity only dopaminergic influences on CIN are shown and cholinergic influences on DAN are omitted except for **(A)**. Dynamic ACh profiles are shown next to each CIN throughout and in a DA profile next to DAN in **(A)** emphasizing the anticorrelated nature of ACh and DA in the healthy dorsal striatum. **(A)** Physiological conditions. Phasic DA elevations are inversely correlated with transient ACh dips creating permissive eligibility windows that result in appropriately scaled plasticity at glutamatergic synapses (blue). DA promotes dSPN LTP via D1 and iSPN LTD via D2. ACh provides counterweighting control via muscarinic receptors (dominant M4 influence in dSPNs opposing D1-driven potentiation; dominant M1 influence in iSPNs promoting dendritic integration/excitability and LTP). **(B)** Parkinsonian OFF state (dopamine-depleted). DA tone is markedly reduced, and cholinergic influence is relatively increased. In established dyskinesia, CIN temporal patterning can be impaired by loss of Kv1-dependent pause competence (“pause-gate failure”), yielding ACh signals that are elevated and less temporally segmented. Net bias favors reduced movement and maladaptive learning. **(C)** Levodopa ON state. Levodopa produces large DA surges that facilitate movement and shift plasticity rules toward dSPN potentiation and iSPN depression, while suppressing ACh output. Repeated, large-amplitude OFF ↔ ON transitions drive metaplasticity and progressive consolidation of dyskinesia-permissive ensembles. **(D, E)** D5 loss/reduced constitutive D5 signaling. Reducing ligand-independent D5 signaling relieves tonic suppression of Kv1-dependent pause generation, restoring pause competence **(D)**. With intact CIN D2s, ON-state DA surges can produce strongly expressed ACh pauses **(E)**, increasing DA–ACh temporal contrast and potentially increasing the efficiency with which DA pulses stamp maladaptive plasticity—one plausible mechanism consistent with enhanced LID reported after global D5 loss. **(F, G)** CIN D2 ablation. In the OFF state **(F)**, CIN D2 loss is predicted to provide limited additional rescue because of predominant pause-gate failure and elevated cholinergic influence can persist via D5/Kv1 constraints. In the ON state **(G)**, DA surges cannot engage CIN D2Rs to impose ACh suppression and/or to enforce tight DA–ACh anticorrelation, yielding higher concurrent ACh tone and/or altered ACh pause timing. This is predicted to re-engage muscarinic counterweights (e.g., M4 braking in dSPNs; M1 counterbalance in iSPNs), reduce the cumulative translation-linked “plasticity load” imposed by repeated levodopa pulses, and attenuate LID—consistent with reduced CIN pERK and blunted ON-associated CIN p-rpS6^240/244^ signals in CIN-D2R knockout mice.

Current motor learning models posit that the anticorrelation between DA and ACh in the striatum creates a permissive coincidence window during which plasticity can occur at corticostriatal synapses (Deffains and Bergman, 2015; Krok et al., 2023; Wieland et al., 2015) (Figure 5A). The anticorrelation of DA and ACh is enhanced and fine-tuned by complex reciprocal facilitatory and inhibitory signaling between DAN and CIN (Figure 5A, central grey plane). SPN, which constitute ~90% of all striatal neurons, fall into two major groups identified as the direct and indirect pathways. “Direct” pathway SPN (dSPN) promote action selection and are excitable by DA via Gα_s/olf_-coupled dopamine 1 receptors (D1), which facilitate long-term synaptic potentiation (LTP) of glutamatergic inputs (Gerfen and Surmeier, 2011; Redgrave et al., 2010; Surmeier et al., 2007; Surmeier et al., 2011; Tritsch and Sabatini, 2012; Tritsch et al., 2012; Zhai et al., 2019) (Figure 5A). Conversely, “indirect” pathway SPN (iSPN) suppress contextually inappropriate actions and are inhibited by DA via Gα_i_-coupled D2, which promotes long-term synaptic depression (LTD); (Gerfen and Surmeier, 2011; Redgrave et al., 2010; Surmeier et al., 2007; Surmeier et al., 2011; Tritsch and Sabatini, 2012; Tritsch et al., 2012; Zhai et al., 2017; Zhai et al., 2019) (Figure 5A).

DA’s effects on SPN are counterbalanced by ACh with dSPN primarily expressing Gα_i_-coupled M4 muscarinic receptors (M4R) and iSPN primarily expressing Gα_s_-coupled M1 muscarinic receptors (M1R), producing LTD and LTP associated responses, respectively (Crittenden et al., 2021; Day et al., 2008; Laverne et al., 2022; Longo et al., 2023; Oldenburg and Ding, 2011; Shen et al., 2007; Shen et al., 2016; Zhai et al., 2023). These reciprocal and opposing effects of DA and ACh on dSPN and iSPN provide a high-contrast mechanism for coordinating acute motor output while simultaneously shaping long-term synaptic plasticity that refines future behavior through learning (Figure 5A) (Krok et al., 2023; Zhai et al., 2025).

In the parkinsonian brain, the degeneration of DAN results in severely reduced DA signaling (Figure 5B, dotted red lines) and a relative increase in ACh signaling as well as increased CIN excitability and loss of the cholinergic pause (Figure 5B, thickened green lines) (Aosaki et al., 1994b; McKinley et al., 2019; Zhai et al., 2025). This shift favors LTD in dSPN, LTP in iSPN and akinesia, producing a loss of skilled movement caused by disturbed motor learning (Cheung et al., 2023). Upon treatment with L-DOPA, DA tone is increased relative to ACh, in part by reinstating D2-mediated inhibition of CIN, and producing a bias toward LTP in dSPN and LTD in iSPN, facilitating movement (Figure 5C) (Aosaki et al., 1994a; Aosaki et al., 1994b; Cheung et al., 2023; Choi et al., 2020; Tubert et al., 2025).

A key factor contributing to the formation of LID is the repeated alternation between the L-DOPA OFF and ON states (Zhai et al., 2025). Repeated OFF–ON transitions during chronic treatment consolidates DA/ACh-dependent plasticity and gradually stabilizes dyskinetic neuronal ensembles in the DLS (Zhai et al., 2025; Girasole et al., 2018). Superimposed on these slow OFF–ON oscillations, CIN also exhibit rapid ‘burst–pause–rebound’ dynamics that define millisecond-scale eligibility windows for synaptic plasticity (Aosaki et al., 1994a; Aosaki et al., 1994b; Apicella et al., 2011; Graybiel et al., 1994; Kimura et al., 1984; Morris et al., 2004; Ravel et al., 1999; Wang et al., 2006). Recent work by Tubert et al. (2025) suggests a mechanistic link between these fast dynamics and the slower state alternations associated with LID. Specifically, CIN pause generation depends on Kv1 conductances, which appear functionally suppressed in established dyskinesia leading to loss of cholinergic pauses in the OFF state. Importantly, this failure to induce pauses in the OFF state is linked to D5 signaling on CIN, and in particular, ligand-independent (constitutive) D5 activity. Consistent with this mechanism, pause competence in the OFF state can be restored by D1/D5 inverse agonism. This observation suggests that along with the hour-long fluctuations in DA/ACh balance over the course of daily L-DOPA treatments, established LID is also associated with the disruption of plasticity mechanisms at the level of individual cholinergic signals spanning milliseconds (Figure 5B, interrupted and thickened green line of the ACh profile). Thus, LID may arise from the interaction of two processes: (1) large-amplitude oscillations between OFF and ON states that bias overall plasticity rules in SPNs, and (2) state-dependent impairments in fast cholinergic pause timing that disrupt synaptic plasticity and promote purposeless movements (Figures 5B,C) (Figge et al., 2024; Zhai et al., 2025). However, it should be noted that dopaminergic control of CIN at the cellular level and how these mechanisms contribute to aberrant plasticity processes remains controversial and incompletely understood.

### 4.2 Reconciling D5 genetics with state/timing mechanisms of LID

Studies of germline D5 ablation mice show that loss of D5 signaling increases LID severity while simultaneously reducing CIN pERK and p-rpS6^240/244^ levels (Castello et al., 2020). These findings are difficult to reconcile with a simple model in which increased CIN activity exacerbates LID. More broadly, reliance on molecular readouts such as pERK and p-rpS6^240/244^ present a persistent challenge in the LID literature, as these markers do not show monotonic relationships with dyskinesia severity across genotypes and manipulations (Bastide et al., 2015). Since these markers reflect translational activity integrated over hours, they may report the intensity of plasticity-related processes rather than instantaneous neuronal activity. In this light, recent results from Tubert et al. (2025) motivate a reappraisal of earlier observations.

In the L-DOPA OFF state, ablation of D5 from CIN, and thus the loss of constitutive D5 activity, would be expected to dampen CIN activity (Figure 5D) (Tubert et al., 2025). In the L-DOPA ON state, along with similar impacts on overall CIN activity, D5 is additionally relevant for triggering the CIN pause on which D2 signaling subsequently acts (Tubert et al., 2025). In this model, the loss of D5 would therefore exacerbate the breakdown of the ACh gate on DA-dependent plasticity by not only reducing general cholinergic tone but also preventing the D2-dependent temporal gating of DA on SPNs by CIN (Figure 5E). Repeated alternations between OFF and ON states, combined with this erosion of ACh gating during the ON state, could therefore bias striatal plasticity toward exaggerated LTP in dSPN and LTD in iSPN, providing a potential mechanism for the enhanced AIMs observed in D5 knockout mice compared to controls (Figure 5E). At the same time, loss of D5 may dampen the magnitude of ACh tone fluctuations across OFF–ON transitions (Figures 5D vs. 5E), potentially aligning with the reduced translational activity reflected in CIN pERK and p-rpS6^240/244^ levels compared with controls (Castello et al., 2020).

### 4.3 CIN D2 as a key node for maintaining the anti-correlation of fast DA–ACh dynamics and enabling meta-plasticity through slow oscillations of state

Within the framework established above, D2 ablation from CIN in the DAN-lesioned striatum should have little effect on physiology during the L-DOPA OFF state compared to controls. The rationale for this is that in the absence of L-DOPA/DA, constitutive D5 activity will drive up cholinergic activity and block the cholinergic pause regardless of CIN D2 presence (Figures 5B,F) (Tubert et al., 2025). However, once in the L-DOPA ON state, mutants with ablation of D2 no longer exhibit D2/Gα_i_-mediated opposition to D5-dependent excitation of CIN (Figure 5G). Thus, the net result of D2 ablation is twofold: (1) There is a relative increase in total ACh and reduced large-scale fluctuations between ON and OFF states, preventing the consolidation of aberrant ACh-dependent plasticity on dSPN and iSPN across ON and OFF alternations (Zhai et al., 2025) and, (2) D2 ablation from CIN switches the anticorrelation of DA and ACh seen in controls treated with L-DOPA to a phase locked positive correlation between ACh and DA transients. This would block DA dependent plasticity on dSPN and iSPN and is consistent with D2’s critical involvement in maintaining plasticity and promoting anticorrelation of DA and ACh fluctuations in the healthy striatum (Figure 5C vs. Figure 5G) (Gallo et al., 2022; Krok et al., 2023; Martyniuk et al., 2022).

In the current study, we find that CIN D2 ablation attenuates LID while blunting CIN pERK and L-DOPA ON-associated CIN p-rpS6^240/244^. Since these markers reflect translational activity integrated over hours, these observations align well with the above interpretation of reduced plasticity due to dampened long and short anticorrelated oscillation of ACh and DA in D2_ChAT_KO mice. More specifically, the increase in CIN p-rpS6^240/244^ observed in controls following repeated L-DOPA dosing could reflect plasticity changes in CIN due to repeated OFF–ON state transitions leading to the recruitment of LID-relevant activity ensembles in the DL striatum. The absence of this increase in mice with D2_ChAT_KO mice suggests that CIN D2 is required for this remodeling program to take place.

### 4.4 Translational strategies

Our data provides evidence that ablating D2 from cholinergic neurons attenuates LID severity and reduces LID-associated molecular markers in CIN. While systemic pharmacological inhibition of D2 would come with unacceptable side effects, there are a number of potential avenues for targeted inhibition of D2 on CIN worth exploring.

The first of these would involve developing transcriptional cis-acting elements that limit gene expression to CIN, allowing local, CIN-specific gene therapy approaches for dampening D2 (Hunker et al., 2025). Another opportunity arises from the observation that CIN exhibit cellular heterogeneity and respond to modulatory signals in a variable manner. In fact, sensitivity to D2 signaling among CIN has been observed to be greatest in the DL striatum (Chuhma et al., 2017; Chuhma et al., 2014), overlapping with localization of LID-associated neuronal ensembles (Girasole et al., 2018). Consistent, we find that the greatest effect of D2 ablation on CIN p-rpS6^240/244^ levels occurs at the lateral edge of the DL striatum (Figure 4). Finally, we previously found that stimulating the GPCR smoothened (Smo) on CIN in the DL striatum attenuates LID, and shortens the cholinergic pause (Malave et al., 2021; Uribe-Cano and Kottmann, 2026). D2 and Smo are both known to localize to the primary cilium of CIN and are likely in close spatial proximity making it feasible to develop pharmacological agents that simultaneously induce Smo agonist and D2 antagonist responses, resulting in increased cellular selectivity and efficacy (Lucarelli et al., 2019; Marley and von Zastrow, 2010; Miyoshi et al., 2014). Interestingly, the primary cilium has also recently been implicated in the majority of sporadic PD cases, (Khan et al., 2021; Khan et al., 2024; Nair et al., 2025; Uribe-Cano and Kottmann, 2024).

### 4.5 Limitations of the current study and testable predictions for follow up experiments

While our data suggest that ablation of D2 from cholinergic neurons attenuates AIMs it is critical to highlight limitations of the current study. Despite replication in two independent cohorts, the overall n is small and only one model of PD was used in these experiments. Clearly, these findings need to be corroborated in additional neurotoxicity and genetic models of PD and LID. Additionally, our p-rpS6^240/244^ immunohistochemical experiments were limited to analysis at the level of individual cells, with low animal replicates. Corroborating these results with additional biological replicates and other methods that parallel p-rpS6^240/244^ quantification is crucial. Further, all CIN of the dorsal and ventral striatum express D2. However, there is evidence that some cholinergic neurons of the globus pallidus (GPe) and Pedunculopontine Tegmental Nucleus (PPT) also express D2. Thus, ablation of D2 driven by ChATCre expression from extra-striatal cholinergic populations could contribute to the attenuation of LID observed here. Notably, our study was also not designed to directly measure the dynamics of L-DOPA OFF–ON transitions, nor fluctuations of ACh and DA *in vivo*, and thus the mechanisms proposed in this discussion remain speculative and in need of hypotheses testing with molecular and behavioral endpoint readouts. Appreciation of the fact CIN D2 acts at multiple subcellular compartments (primary cilia vs. extra synaptically), which we have not disentangled here, would also greatly benefit future studies. Finally, direct causal links between CIN D2 loss, D5/Kv1 pause competence, and SPN plasticity mechanisms across L-DOPA OFF and ON states remain to be established and are highly speculative.

However, what emerges from our attempts to integrate our findings into recently updated proposals of striatal function and dysfunction are several testable predictions of feasible follow up studies. First, if D5/Kv1 signaling constrains pause competence in dyskinesia, CIN-D2 ablation may not restore OFF-state pauses but could alter pause structure during the L-DOPA ON state or modify responses to D1/D5 inverse agonism. Second, D2_ChAT_KO mice should exhibit reduced slow ON–OFF oscillations in ACh across the L-DOPA dosing cycle which could be monitored using genetically encoded ACh sensors. Third, ablation of CIN D2 may weaken the millisecond-timescale organization of DA bursts and ACh pauses during the L-DOPA ON state, thereby preventing maladaptive eligibility windows for synaptic plasticity. Fourth, CIN D2 ablation may attenuate state-dependent remodeling of SPNs associated with LID, particularly within the DL striatum. Finally, if p-rpS6^240/244^ reflects the cumulative intensity of plastic change, its magnitude should correlate more closely with cumulative LID severity across treatment days than with instantaneous cholinergic tone.

While these ideas remain to be tested, they place CIN D2 signaling within a framework linking DA–ACh coordination, state-dependent plasticity, and dyskinesia. In this view, D2 receptors in CIN may represent a tractable molecular entry point for strategies aimed at preserving the therapeutic benefits of L-DOPA while limiting its debilitating motor side effects.

## Data Availability

The raw data supporting the conclusions of this article will be made available by the authors, without undue reservation.
