# Supplementary material for: Dopamine 2 receptor ablation from cholinergic neurons attenuates L-DOPA induced dyskinesias
Source: Front Aging Neurosci. 2026 May 12;18:1779861. doi: 10.3389/fnagi.2026.1779861 (PMC13201114; doi:10.3389/fnagi.2026.1779861)
Supplement: Supplementary file 2 [file Data_Sheet_1.pdf]

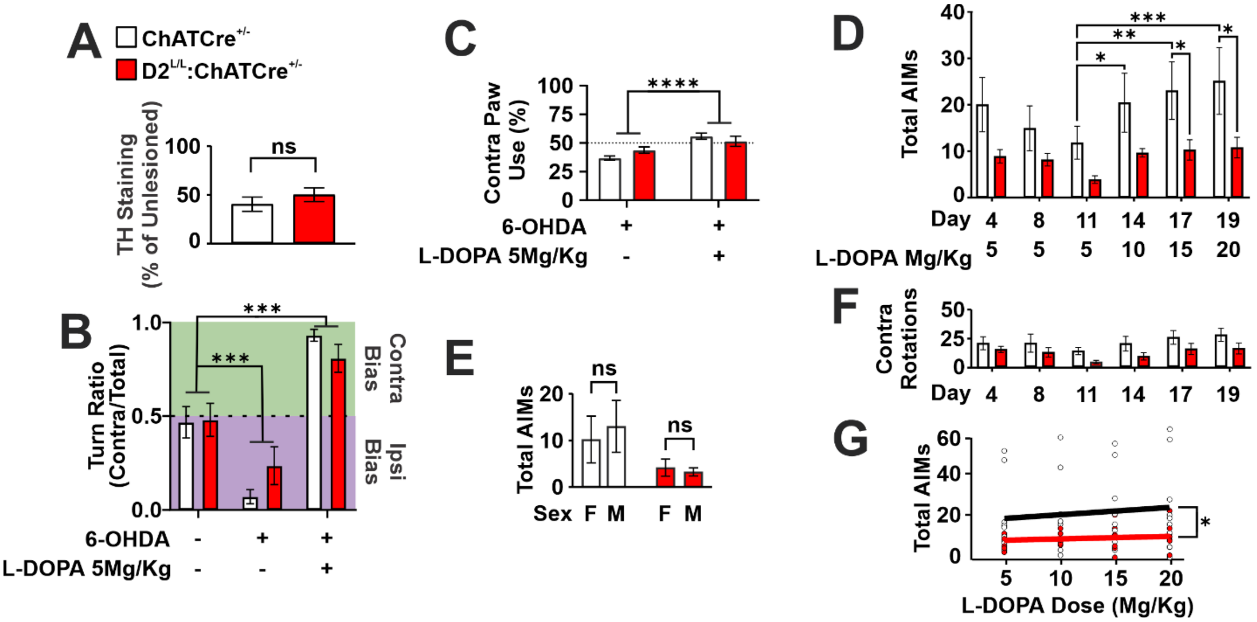

Supplementary Figure 1 (A) Quantification of tyrosine hydroxylase (TH) reduction in the dorsal striatum of animals from the second replication experiment following 6-hydroxydopamine (6-OHDA) injection. Values are expressed as the ratio of lesioned/unlesioned staining intensity in ChATCre$^{+/-}$ controls or D2$^{L/L}$:ChATCre$^{+/-}$ animals ($n = 8–9$; unpaired two-tailed Student's t test, $p > 0.05$). **(B)** Changes in turn bias in animals from the second replication experiment, measured at baseline, after unilateral 6-OHDA lesion, and after subsequent L-DOPA treatment (5Mg/Kg). Turn bias was quantified as the ratio of contralateral turns over total turns ($n = 8–9$ per genotype; two-way repeated-measures ANOVA: Timepoint effect, $F(1.795, 26.92) = 60.24$, $p < 0.0001$; Genotype effect, $F(1, 15) = 0.07$, $p > 0.05$; Timepoint × Genotype interaction, $F(2, 30) = 2.43$, $p > 0.05$; post hoc Tukey's multiple comparisons test: ***$p < 0.001$). **(C)** Contralateral paw use in unilateral 6-OHDA animals from the second replication experiment, measured before and after L-DOPA treatment. Data represent the percentage of total rears performed with the forelimb contralateral to the lesion ($n = 8–9$ per genotype; two-way repeated-measures ANOVA: Timepoint effect, $F(1, 15) = 31.52$, $p < 0.0001$; Genotype effect, $F(1, 15) = 0.12$, $p > 0.05$; Timepoint × Genotype interaction, $F(1, 15) = 5.59$, $p < 0.05$). **(D)** Replication of total abnormal involuntary movement (AIM) scores across 19 days of L-DOPA treatment ($n = 8–9$ per genotype; two-way repeated-measures ANOVA: Day/Dose effect, $F(5, 75) = 3.98$, $p < 0.01$; Genotype effect, $F(1, 15) = 3.92$, $p > 0.05$; Genotype × Day/Dose interaction, $F(5, 75) = 0.60$, $p > 0.05$; post hoc Tukey's multiple comparisons test: *$p < 0.05$, **$p < 0.01$, ***$p < 0.001$). **(E)** Total AIM scores at 5Mg/Kg L-DOPA, grouped by sex and genotype ($n = 4–6$ per condition; two-way ANOVA: Sex effect, $F(1, 13) = 0.05$, $p > 0.05$; Genotype effect, $F(1, 13) = 3.62$, $p > 0.05$; Sex × Genotype interaction, $F(1, 13) = 0.20$, $p > 0.05$). **(F)** Total contralateral rotations recorded across the same 19 days of L-DOPA treatment shown in Panel (D) ($n = 8–9$ per genotype; two-way repeated-measures ANOVA: Day/Dose effect, $F(2.160, 32.41) = 3.36$, $p < 0.05$; Genotype effect, $F(1, 15) = 2.97$, $p > 0.05$; Genotype × Day/Dose interaction, $F(5, 75) = 0.14$, $p > 0.05$). **(G)** AIM severity plotted against L-DOPA dose for each genotype in the second replication experiment. Slope differences between genotypes were assessed by ANCOVA ($n = 8–9$ per genotype; $F(1, 4) = 12.13$, $p < 0.05$).

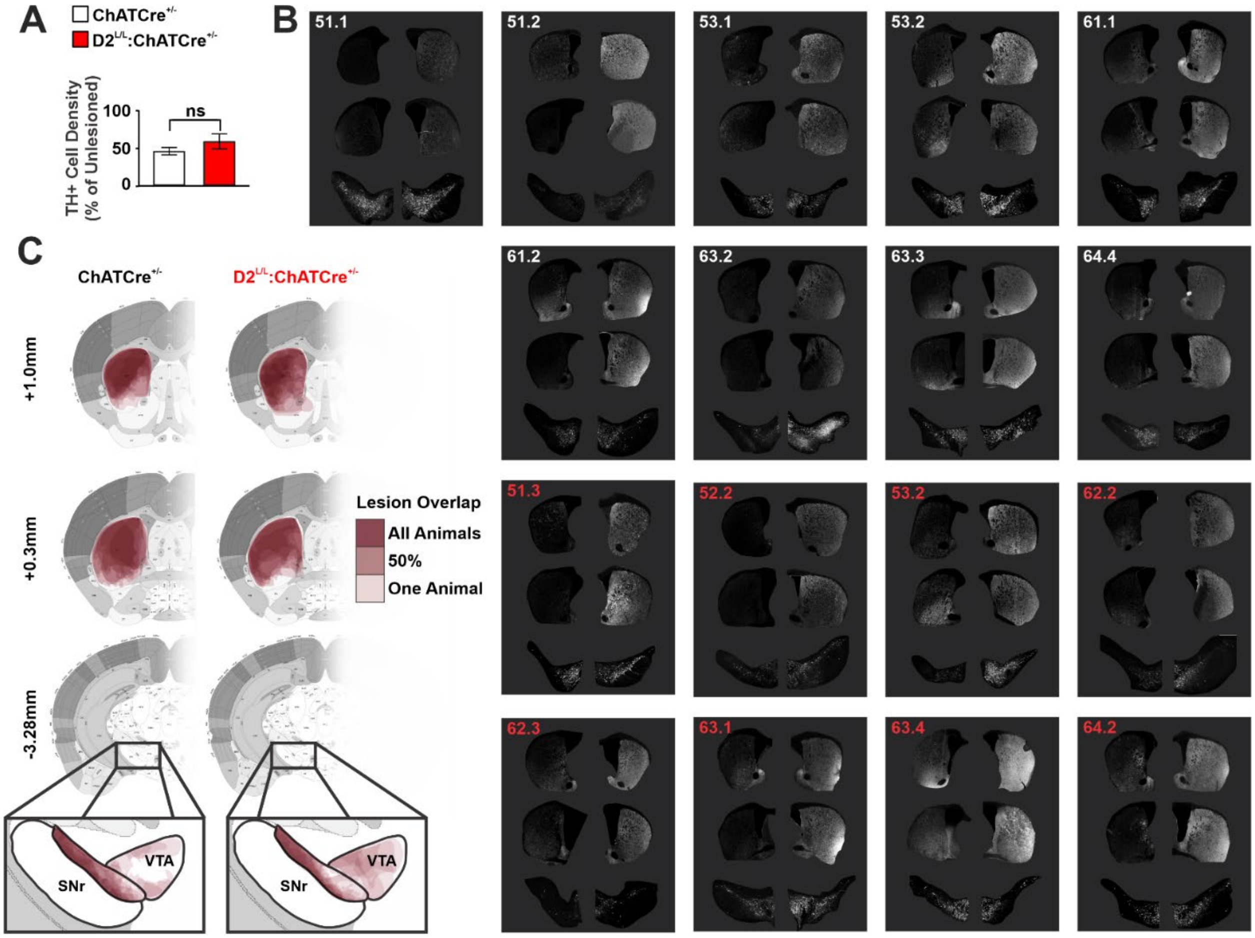

Supplementary Figure 2 (A) Quantification of tyrosine hydroxylase-positive (TH^{+}) cell body density in the substantia nigra pars compacta, reported as a percentage of the unlesioned hemisphere (n = 8–9 per genotype; unpaired two-tailed Student's t test, $p > 0.05$). **(B)** Representative images of TH immunoreactivity in the striatum and midbrain for all animals included in the replication experiment. For each animal, the left image represents the 6-OHDA-treated hemisphere. ID label color indicates genotype (White = $ChATCre^{+/-}$; Red = $D2^{L/L}$:$ChATCre^{+/-}$). **(C)** Anatomical mapping of 6-OHDA lesion overlap across all animals in each genotype. Regions of TH loss were identified across multiple striatal and midbrain sections by thresholding images to retain the top 10% of pixel intensities. Lesion regions of interest (ROIs) were defined as areas lacking abundant high-intensity TH+ pixels. Individual red contours indicate the ROI of reduced TH intensity for each animal at a given anterior-posterior plane. The color scale reflects the percentage of animals within each cohort showing TH loss at a given anatomical location. ROIs from $ChATCre^{+/-}$ controls are shown on the left, and those from $D2^{L/L}$:$ChATCre^{+/-}$ animals are on the right.
